# Speaking up culture of medical students within an academic teaching hospital: Need of faculty working in patient safety

**DOI:** 10.1371/journal.pone.0222461

**Published:** 2019-09-12

**Authors:** David Schwappach, Gerald Sendlhofer, Lars-Peter Kamolz, Wolfgang Köle, Gernot Brunner

**Affiliations:** 1 Swiss Patient Safety Foundation, Zurich, Switzerland; 2 Institute of Social and Preventive Medicine, University of Bern, Bern, Switzerland; 3 Executive Department for Quality and Risk Management, University Hospital Graz, Graz, Austria; 4 Research Unit for Safety in Health, c/o Division of Plastic, Aesthetic and Reconstructive Surgery, Department of Surgery, Medical University of Graz, Graz, Austria; 5 Department of General Otorhinolaryngology, Medical University of Graz, Graz, Austria; 6 Medical Directorate, University Hospital Graz, Graz, Austria; University of Adelaide, AUSTRALIA

## Abstract

**Background:**

Speaking up behavior is a manifestation the culture of safety in an organization; however, withholding voice is commonly observed. Within one academic teaching hospital, it was the aim to assess students’ speaking up behaviors and perceived culture in order to stimulation of the academic development in terms of patient safety.

**Methods:**

Survey amongst medical students using a validated questionnaire. Data were analysed using descriptive statistics.

**Results:**

326 individuals completed the questionnaire (response rate 24%). 37% of responders were in their 5^th^- 6^th^ clinical term, 32% were in their 7^th^-8^th^ term and 31% were in the 9^th^-12^th^ term. 69% of students had a specific safety concern in the past four weeks, 48% had observed an error and 68% noticed the violation of a patient safety rule. Though students perceived specific patient safety concerns, 56% did not speak up in a critical situation. All predefined barriers seemed to play an important role in inhibiting students’ voicing concerns. The scores on the psychological safety scale were overall moderately favourable. Students felt little encouraged by colleagues and, in particular, by supervisors to speak up.

**Conclusion:**

Speaking up behaviour of students was assessed for the first time in an Austrian academic teaching hospital. The higher the term the more frequent students reported perceived patient safety concerns or rule violations and withholding voice. These results suggest the need to adapt the curriculum concept of the faculty in order to address patient safety as a relevant topic.

## Introduction

Speaking up can be defined as assertive communication of quality and patient safety concerns by a team member through information, questions, or opinions in situations where clinical rules are neglected, forgotten or even unknown by a healthcare professional (HCP) in order to avoid patient harm [1–9]. Effective teamwork and non-technical skills are essential for the reduction of medical errors [10]. Speaking up by HCPs to their colleagues is increasingly acknowledged as an important way to reduce risks [8, 11,12]. However, withholding voice is common and was confirmed recently within our university hospital [8]. More than half of HCPs perceived specific concerns about patient safety at least once in the last four weeks, however more than 40% remained silent in a critical situation [8]. This HCPs’ silence or withholding voice may have many reasons and the existence of steep hierarchies within HCPs may be the most dominant [10].

Within our academic teaching hospital, more than 7.000 employees care for patients and more than 3.000 employees are responsible for science and teaching. As medical students have to attend several clinical internships in their academic career, they become automatically junior members of care teams and may therefore also play an important role in supporting patient safety [10]. In 2012 a study in Australia revealed that more than 80% of students were willing to speak up towards others students, however, this number decreased for those who were willing to do so to HCPs [10]. Reasons for not speaking up amongst students had been reluctance to question senior staff, unwillingness to interrupt and embarrassment [10,13]. A nursing study also showed that during internships students were frequently asked to condone poor practice [14].

It is obvious that courage is needed to speak up as it demands the readiness to stand up and to do the right thing at the right time [15,16]. However, it is also known that students diminish their role during internships to that of being “just a student” [17]. Students are, to a certain degree, in a bind. On the one hand, they have to complete and pass the internships and, on the other hand, they are evaluated by HCPs. Speaking up could therefore cause a conflict for medical students as they are alerting a doctor to non-compliance of a clinical rule [18]. And most important, a safety culture needs to be in place giving students and HCPs the certainty to do so.

So far, in German speaking countries no data are available concerning speaking up behaviors of medical students, in particular, in relation to their study progress and years of training. To assess the speaking up behavior and perceived culture in one academic teaching hospital in Austria a validated questionnaire was used to assess self-reported speaking up related behaviors, climate and perceived barriers. Results may give potential implications on the development of a patient safety curriculum.

## Materials and method

The Ethics Committee of Medical University of Graz approved the study (vote#: 30–303 ex17/18).

### Study population

A cross-sectional survey study was conducted among medical students doing their clinical terms at one large Austrian academic teaching hospital. The diploma in human medicine prepares students for their future professions as doctors in all specialties. Theoretical and practical skills are taught in an integral, subject-centred and patient-oriented way. The first part of the human medicine study (four terms) provides knowledge and basic understanding of the human organism. Essential components into general medicine, insight into nursing in the context of a ward internship, which ensures early patient contact are part of the first term. In the second part of the human medicine study (six terms) students develop the knowledge about the healthy and sick organism. It is based on topic-centered, patient-centered, interdisciplinary teaching, including clinical presentations and using new forms of teaching such as problem-based learning. Increasing training of medical skills and abilities continuously ensures the practical application of theoretically acquired knowledge. Students have to complete clinical terms for at least of 18 weeks during the second part of the study. Each clinical term lasts for four weeks and they rotate between units. Finally, during the clinical practical year (2 terms) students are actively integrated as learning team members in everyday clinical practice. In total, students must be clinically active for 48 weeks in a teaching hospital. They deepen and acquire the competences listed in the “Austrian competence level catalogue” for medical skills in accordance with European requirements [19].

In the present study, students in their 5^th^ to 12^th^ term were included and invited by the Organisational Unit for Studies and Teaching to participate via an online survey. It could have happened that not all students between the 5^th^ and 10^th^ term were recently exposed to a four weeks clinical term. They were instructed that their responses were collected anonymously and used only in aggregate. The survey was open for four weeks. As proposed by Burns et al., weekly reminders were automatically sent by the system to non-responders in order to increase the response rate [20]. There was no sample size estimation as the study had an explorative character, solely.

### Survey instrument and measures

We used the ‘**S**peaking **U**p about **P**atient **S**afety **Q**uestionnaire (SUPS-Q)’. Development and psychometric properties of this survey have been reported in detail [21]. The instrument was originally developed in Switzerland and has been applied to health care staff in diverse hospitals [1, 11]. For this study, the Austrian version of the SUPS-Q was used [8]. Few minor language adjustments were made to ensure that the survey fits the clinical context of medical students. In particular, questions addressing personal data were omitted, e.g., occupational group and clinical function. A pre-test among 100 medical students revealed no problems (e.g., no excessive missing data or ceiling effects). Qualitative content analysis of open-ended responses were categorized using the software program “MAXQDA 12”. Of all open-ended responses, some outstanding comments were translated into English. If names or units where mentioned, they were deleted.

The SUPS-Q is a brief questionnaire that assesses health care workers’ perceived patient safety concerns, their past speaking up behaviors, perceived barriers for speaking up, their evaluations of the speaking up climate at their workplace and their anticipated speaking up behavior.

The frequency of speak up-related behaviours includes three scales addressing 1) the frequency of perceived safety concerns (3 items), 2) the frequency of withholding voice, i.e., not to speak up in specified situations (4 items) and 3) the frequency of speaking up (4 items). Response options for the items in these scales are anchored to “in the last four weeks” and include “never” (0 times in the last 4 weeks), “rarely” (1–2 times), “sometimes” (3–5 times), “often” (6–10 times) and “very often” (more than 10 times during the last 4 weeks). Thus, higher mean scale values indicate higher frequencies of past speaking up and withholding voice behaviours respectively.

Barriers for speaking up are assessed with one multiple choice item asking for the relevance of 6 predefined barriers in bringing up patient safety concerns (yes/no).

Speak up-related climate is assessed by 11 items organized in 3 sub-scales: 1) the psychological safety for speaking up scale (5 items), 2) the encouraging environment for speaking up scale (3 items) and 3) the resignation scale (3 items). The answers are coded in a 7-point-Likert scale from “strongly disagree with this statement” to “strongly agree with this statement”. Thus, higher mean scale scores indicate higher levels of perceived psychological safety at workplace, higher levels of perceiving the workplace as encouraging speaking up and higher levels of resignation with speaking up respectively. A high level of resignation is indicative of a poorer safety culture. Survey items are presented in the tables in this manuscript.

The survey also covers a clinical vignette that describes a hypothetical speaking up situation. The vignette reads “You are on a daily round with several doctors and nurses. During the round, the attending doctor shakes hands with a patient who recently had surgery. He wants to examine the patient’s wound. However, the attending does not apply gloves and/or does not disinfect their hands.” Responders were instructed to consider their anticipated behaviours if they would find themselves in the situation. They were asked to complete four questions addressing the realism of the situation, the potential for patient harm, their discomfort with and likelihood of speaking up. These questions each used a 1–7 response scale with specifically labelled poles. The survey closes with an open-end question inviting responders to provide feedback, personal experiences with speaking up and comments.

### Statistical analysis

Descriptive statistics (mean, standard deviation (SD)) are reported for all items. For items organized in scales, mean scale scores were computed as aggregate measures. Cronbach’s alpha was computed as a measure of scale reliability. To determine associations of survey responses with study term (i.e., length of experience in the hospital setting) nonparametric test for trend across terms and analysis of variance (ANOVA) were used as appropriate. We used the nonparametric test for trend across ordered groups developed by Cuzick, which is an extension of the Wilcoxon rank-sum test. A correction for ties is incorporated into the test [22]. Regression analysis was conducted to model students’ reported likelihood to speak up in the situation described in the vignette (i.e., the missed hand disinfection) as outcome. Students’ rating of realism of the situation, rating of potential of harm for the patient, past withholding voice and past speaking up behaviours, their evaluation of the speak up related climate at the hospital, and their current term of studies were included as independent variables. All tests were two-sided and a p-value < 0.05 was regarded statistically significant. All analyses were conducted using STATA V14.

## Results

Of invited students (n = 1.524), 326 individuals completed the questionnaire (response rate 24%). At the time of survey participation, 37% of responders were in their 5^th^- 6^th^ clinical term, 32% were in their 7^th^-8^th^ term and 31% were in the 9^th^-12^th^ term. Of participating students, 53% were females. The largest fraction of responders spent their last training in the department of surgery (36%), followed by internal medicine (15%), pediatrics (8%), orthopedics and traumatology (7%), anesthesiology and intensive care (6%), gynecology and obstetrics (6%), and neurology (6%).

### Safety concerns and speaking up behaviors

Students commonly reported perceived patient safety concerns as shown in Table 1. For example, more than two thirds experienced safety rule violations by colleagues at least once during the last four weeks. Frequency of safety concerns was significantly associated with higher clinical term. Among students in their 5^th^-6^th^ term, 64% responded they had not observed a potentially harmful error during the past four weeks whereas it were 41% of students in their 9^th^-12^th^ term (test for trend p<0.001). Rule violations were observed at least once during the past four weeks by 57% of students in their 5^th^-6^th^ term and by 78% of students in their 9^th^-12^th^ term (test for trend p = 0.002). The majority of students reported at least one episode of withholding voice despite safety concerns during the past four weeks (Table 1). A trend of remaining silent was seen for students of higher terms. For example, the fraction of students reporting they had chosen not to bring up specific concerns about patient safety was 48%, and 68% of students in their 5^th^-6^th^ and 9^th^-12^th^ term, respectively. Nearly half responders reported at least one episode of speaking up in the past four weeks (Table 1).

**Table 1:** Count (relative frequencies) of self-reported perceived patient safety concerns, withholding voice and speaking up behaviours ^A^.

	never	rarely	sometimes	often	very often	
***In everyday work*, *it sometimes happens that things go wrong and risks to patients arise*. *This could be as a result of medication error*, *poor hand hygiene or missing documentation*. *Over the last 4 weeks*, *how frequently…***						
*Perceived concerns (Cronbachs Alpha = 0*.*78)*						
*… have you had specific concerns about patient safety*?	107 (33%)	139 (43%)	51 (16%)	17 (5%)	10 (3%)	***
*… have you observed an error which—if uncaptured—could be harmful to patients*?	171 (52%)	118 (36%)	29 (9%)	7 (2%)	1 (0%)	***
*… have often have you noticed that your workplace colleagues haven’t followed important patient safety rules*, *intentionally or unintentionally*?	104 (32%)	108 (33%)	78 (24%)	21 (6%)	13 (4%)	**
*Withholding voice (Cronbachs Alpha = 0*.*90)*						
*… did you choose not to bring up your specific concerns about patient safety*?	126 (39%)	93 (29%)	54 (17%)	31 (10%)	20 (6%)	**
*… did you keep ideas for improving patient safety in your unit to yourself*?	126 (39%)	84 (26%)	60 (18%)	39 (12%)	16 (5%)	*
*… did you remain silent when you had information that might have prevented a safety incident in your unit*?	186 (57%)	85 (26%)	32 (10%)	12 (4%)	10 (3%)	
*… did you not address a colleague (doctors and/or nurses) if he/she didn’t follow important patient safety rules*, *intentionally or unintentionally*?	150 (46%)	85 (26%)	44 (14%)	24 (7%)	21 (6%)	*
*Speaking up (Cronbachs Alpha = 0*.*86)*						
*… did you bring up specific concerns about patient safety*?	172 (53%)	103 (32%)	38 (12%)	7 (2%)	3 (1%)	**
*… did you address an error which–if uncaptured–could be harmful for patients*?	182 (56%)	89 (28%)	42 (13%)	6 (2%)	4 (1%)	
*… did you address a colleague (doctors and/or nurses) when he/she didn’t follow important patient safety rules*, *intentionally or unintentionally*?	188 (58%)	90 (28%)	32 (10%)	9 (3%)	3 (1%)	
*… did you prevent an incident from occurring as a consequence of bringing up specific concerns about patient safety*?	242 (77%)	59 (19%)	12 (4%)	3 (1%)	0 (0%)	

^A^ Categories were presented as: “never” (0 times in the last 4 weeks), “rarely” (1–2 times), “sometimes” (3–5 times), “often” (6–10 times) and “very often” (more than 10 times during the last 4 weeks

* nonparametric test for trend across clinical terms p<0.05,

** p<0.01,

*** p<0.001

**Fig 1:**
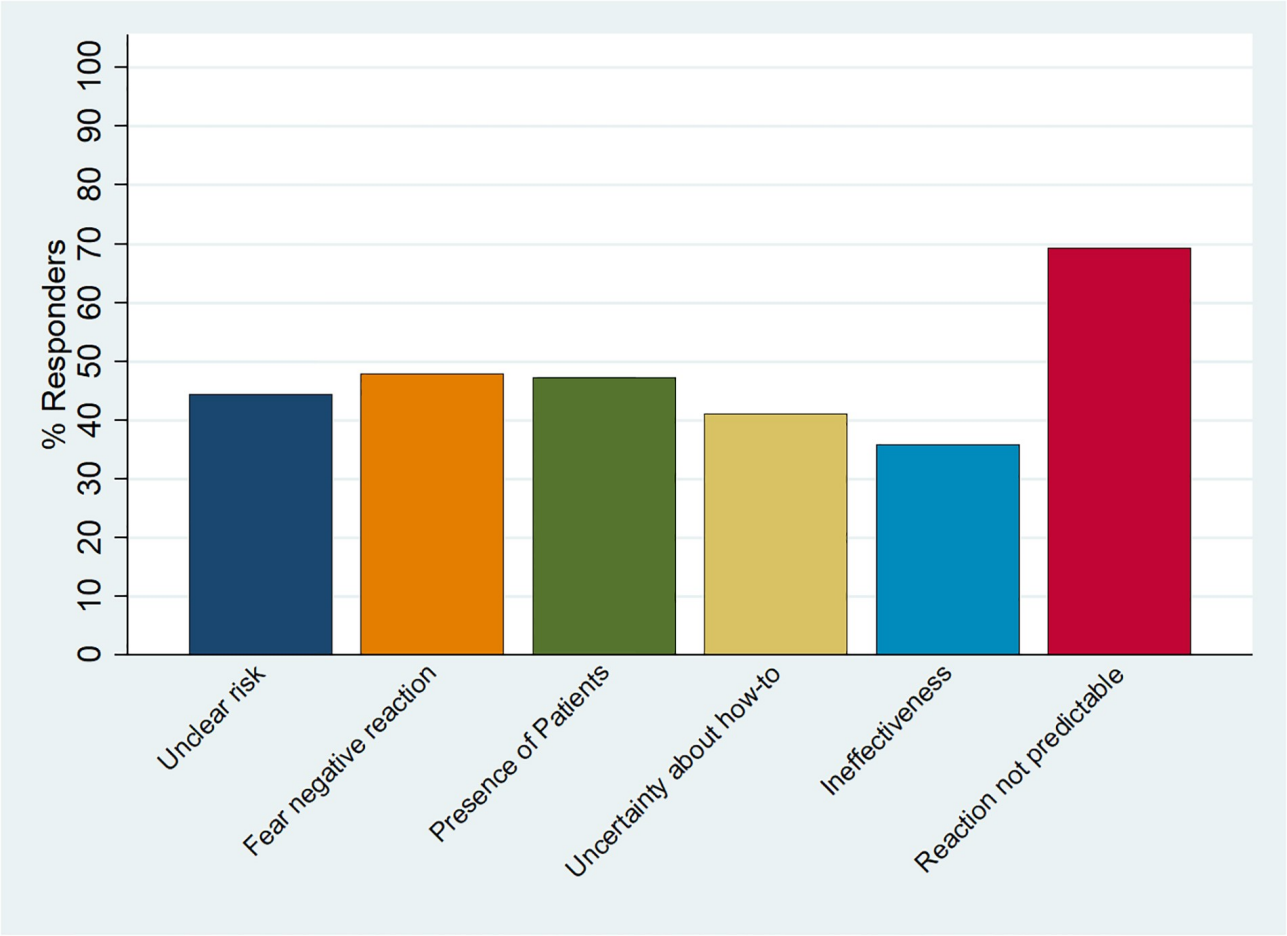
Frequency of relevant barriers for bringing up patient safety concerns.

All predefined barriers seem to play an important role in inhibiting students’ voicing concerns (Fig 1). The reason cited most often was “reaction of the person causing concern is not possible to predict”, indicated by 69%. “Lack of clarity about the risks of a situation”, “fear of negative consequences”, “presence of patients or relatives” and “ineffectiveness of speaking up (it does not make a difference)” were mentioned as factors inhibiting speaking up by 36%–48% of students. Reported barriers were not associated with study term.

### Speaking up related climate

Table 2 reports the results of the speak up related climate items. The scores on the psychological safety scale were overall moderately favourable. However, there was only a low level of support for the statement that “the culture in my unit/clinical area makes it easy to speak up about patient safety concerns.” In addition, students felt little encouraged to speak up by colleagues and, in particular, by supervisors. The mean score on the “encouraging environment for speaking up” scale of 3.1 (SD 1.6) is low. Contrary, levels of resignation were already substantial in students (mean score of the “resignation towards speaking up” scale: 3.6 (SD 1.4)). There were no differences in mean responses related to study term on the item and on the scale level, with one exception. Students in their 9^th^-12^th^ term were more likely to agree with the statement “when I have concerns regarding patient safety, it is difficult to submit them” (mean 5^th^-6^th^ term: 3.1 (SD 1.7); mean 7^th^-8^th^ term: 2.9 (SD 1.7); mean 9^th^-12^th^ term: 3.7 (SD 1.9); p = 0.0022).

**Table 2:** Mean scores on a 7-point Likert scale and standard deviations for speak up related safety climate items, scales and total scale score ^A^.

Items organized in scales	Mean	(SD)	p^B^
Psychological Safety for Speaking up scale (α = 0.86)	4.4	1.3	0.30
I can rely on my colleagues (physicians and/or nurses), whenever I encounter difficulties in my work.	5.1	1.5	0.28
I can rely on the shift supervisor (person in charge of a shift) whenever I encounter difficulties in my work.	5.0	1.6	0.43
The culture in my unit/clinical area makes it easy to speak up about patient safety concerns.	3.8	1.8	0.57
My colleagues (physicians and/or nurses) react appropriately, when I speak up about my concerns about patient safety.	4.2	1.5	0.33
My shift supervisors (person in charge of a shift) react appropriately, when I speak up about my patient safety concerns.	4.1	1.6	0.60
Encouraging Environment for Speaking up scale (α = 0.85)	3.1	1.6	0.29
In my unit/clinical area, I observe others speaking up about their patient safety concerns.	3.3	1.8	0.77
I am encouraged by my colleagues (physicians and/or nurses) to speak up about patient safety concerns.	3.1	1.8	0.17
I am encouraged by my shift supervisor (person in charge during a shift) to speak up about patient safety concerns.	2.9	1.8	0.08
Resignation towards Speaking up scale (α = 0.66)	3.6	1.4	0.35
Having to remind staff of the same safety rules again and again is frustrating.^1^	4.2	1.9	0.77
Sometimes I become discouraged because nothing changes after expressing my patient safety concerns.^1^	3.4	1.7	0.68
When I have concerns regarding patient safety, it is difficult to submit them.^1^	3.2	1.8	<0.01
Total scale (α = 0.87)	4.0	1.2	0.47

^1^Negatively worded items, recoded for the total scale score.

^A^ Response options ranged from 1 (strongly disagree) to 7 (strongly agree)

^B^ One way analysis of variance for differences in mean ratings between students of different clinical terms

### Vignette

**Fig 2:**
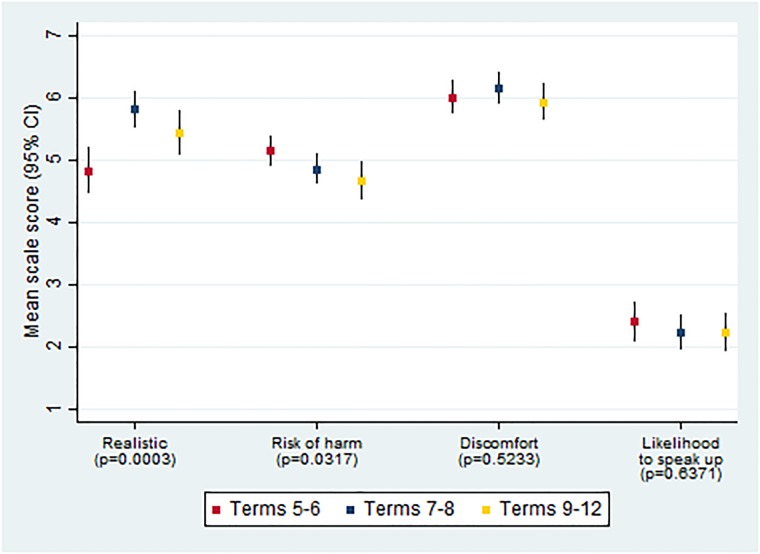
Mean ratings and 95% CI of the missed hand disinfection vignette by study term. Reported p-values for differences in means between study terms (ANOVA).

When confronted with the missed hand disinfection vignette, most students rated this situation as realistic (mean score 5.3, SD 1.8) and as posing a severe risk to the patient (mean score 4.9, SD 1.3). Students reported a high level of discomfort to speak up in the described situation (mean score 6.0, SD 1.4) and–on average—anticipated their likelihood to speak up as low (mean score 2.3, SD 1.6), despite the perceived potential harm for patients. Anova analysis revealed significant differences in perceived realism and potential for harm relative to study term, but not in level of discomfort and likelihood to speak up (Fig 2).

Table 3 reports the results of the regression analysis with likelihood to speak up as outcome in the vignette. Higher perceived risk of harm for the patient, past episodes of speaking up and a more favourable climate score were all significantly associated with a higher self-reported likelihood to speak up. Contrary, higher ratings of realism of the situation and a history of withholding voice behaviours decreased the self-reported likelihood that students would speak up. After accounting for these factors, study term was not independently related to self-reported likelihood to speak up.

**Table 3:** Results of multiple regression analysis with reported likelihood to speak up as outcome.

Variable	Coefficient	95% CI	p
Rating of realism (range: 1–7)	-0.208	-0.297,-0.118	<0.001
Risk of harm rating (range: 1–7)	0.220	0.109,0.331	<0.001
Past withholding voice behavior (range: 1–5)	-0.344	-0.518,-0.169	<0.001
Past speaking up behavior (range: 1–5)	0.503	0.276,0.729	<0.001
Total climate score (range: 1–7)	0.292	0.145,0.440	<0.001
Clinical term (to base: 5^th^-6^th^ term)			
7^th^-8^th^ term	0.204	-0.166,0.574	0.278
9^th^-12^th^ term	0.085	-0.287,0.458	0.653
Constant	0.982	-0.161,2.125	0.092
R2	0.31		
Overall model p	<0.001		
n	317		
Cohen’s f2	0.442		

### Feedback of students

In sum, students gave 90 open-ended responses. Students’ free text feedback was summarized in two main categories: 1) experiences of students and 2) solutions (Table 4). Within the first category, hierarchy was the most commonly mentioned challenge for speaking up (mentioned by n = 11). Further challenges were unknown consequences (n = 4), fear of verbal attacks of HCPs who received speaking up (n = 3), the dependence of students on their mentors (n = 2) or the violation of polite manners (n = 2). Students also reported positive (n = 6) and negative experiences (n = 16) of patient safety relevant occurrences. In category two, all answers were clustered concerning solutions to improve the speaking up culture. Most often more courageous and more active feedback were mentioned (n = 5). Furthermore, training on patient safety and safety culture was proposed (n = 3).

**Table 4:** Selection of students’ responses clustered in two categories: 1) experiences of students and 2) solutions.

**Experiences of students**
*"No student would ever dare to speak up to a doctor on his failure*.*"*
*"My biggest concern when addressing failures to hierarchically higher people is to make them feel embarrassed and to receive bad treatment/teaching as a response*. *Productive criticism is immediately counted as an attack on one’s own person*. *Students may have even better ways to criticize*, *since the worst thing that happens to them may be the charge of incompetence (which is true)*.*"*
*"There are (especially in one discipline) a lot of bosses and senior physicians who feel beyond any doubt and verbally attack students and others"*
*"My concrete experience was that colleagues often did not wear the necessary protective clothing due to time constraints before entering respective rooms*, *but gloves were always worn and hand disinfected*.*"*
*"Furthermore*, *I noticed that that there are well written specifications / elaborated procedures for a variety of events but these procedures are simply not implemented in practice*.*"*
*"That the handling in a specific outpatient ward*, *both with patients and with the students was very rude*, *questions and remarks were ignored*.*"*
*"But then I decided to say*: *Attention*, *senior doctor*: *you touched at the x-ray machine with your elbow*! *And he said*:*" What me*? *Really*? *"First he was a bit grumpy and everyone was surprised that I said that but when he put on his new sterile clothes he said loudly in the room*: *Dear colleagues*: *take this student as a role model*! *Something like that is rare*! *And after the surgery*, *he thanked me once again for speaking up*, *which was a really nice moment for me*!*"*
*“All very nice and careful on patient safety and friendly*!*”*
*"Young colleagues perform very well in hand hygiene disinfection and there is nothing to complain about*.*"*
**Solutions**
*"Communication is very important for effective teamwork and patients*. *For patient safety it should be possible (without hesitation) to also give advice*.*"*
*"Concerning patient safety I miss an in-depth*, *scientifically accurate and clinically relevant introduction to the topic during the study*. *There is a seminar on "quality management”*, *however it is in my opinion currently didactically very poorly designed*, *also the subject of patient safety is not addressed here—that should change*! *"*
*"Speaking-up would work in my opinion in a subtle way (with gestures) or by indirect addressing*, *for example by saying" for self-protection*…"-"
*More compulsory error handling seminars should be introduced*. *It should not just include the avoidance of mistakes*, *but also to raise awareness that we are going to make mistakes and how we handle them without putting too much mental strain on us*.
*“As a student*, *concerns (especially regarding hand hygiene) were not taken serious*, *and cannot be applied in strictly hierarchically organized wards*. *The longer I work at the same ward*, *the sooner I would speak up for improvements*.*”*

## Discussion

This study is the first to report speaking up behaviours and climate for medical students in one Austrian academic teaching hospital using the validated SUPS-Q in German speaking countries. Overall, medical students reported perceived patient safety concerns, which increased significantly with students’ term. Furthermore, rule violations were also observed more often by students of higher terms. Although students perceived patient safety concerns and rule violations, they remained silent, which was more obvious in students of higher terms. The majority reported that an important role in inhibiting students’ voicing concerns was the reaction of the person causing a concern is not possible to predict. All predefined barriers seemed to also play an important role in withholding voice. Students felt also less encouraged to speak up which was associated with a missing safety culture in the clinical departments during their internships. It was raised that it is difficult to submit patient safety concerns. Students considered the described vignette to be a realistic scenario in a healthcare setting; however, the likelihood to speak up was low and was accompanied by a high level of discomfort to speak up.

67% of students had at least once a specific concern about patient safety in the past four weeks, 48% had observed an error and 68% noticed the violation of a patient safety rule. Though students perceived specifics patient safety concerns, 56% did not speak up in a critical situation during the last four weeks. Perceived concerns of students were similar to that of surveyed HCPs the year before using the same survey instrument: 69% of HCPs had at least once a specific concern in the past four weeks, 57% had observed an error, 58% noticed HCPs who ignored a patient safety rule, but 42% remained silent [8]. It was also obvious in the latter study that HCPs gave a socially desirable response as nearly 100% of HCPs reported having spoken up. Results of the present study put the previous in a different light. Students are attentive observers and, as student’s feedback indicated, that the everyday life in the hospital is characterized by hierarchical structures, they fear to speak up. Reasons for not speaking up might be that the psychological safety for speaking up was only moderately expressed, accompanied by a less encouraging environment and substantial resignation. Regression analysis showed that the perceived climate influenced if students wanted to speak up. It was demonstrated that withholding voice and speaking up perpetuate. Students who withhold their voice earlier remain to their habit, whereas students who already spoken up would rather do it again. Reasons might be that the experience of speaking up was either not so bad or students were confident.

The vignette was considered as a realistic scenario and was with increasing term even more realistic. However, the higher the term the lower the risk of harm was estimated. Compared to the previous study, the vignette was scored twice as much with regard to the dangerousness of the scenario [8]. However, high-perceived risk for the patient the likelihood of students to speak up was very low and was accompanied with a very high discomfort to do so. Students’ reasons for discomfort ranged from barriers such as presence of the patient, reaction of the HCPs and fear of negative reaction as well as ineffectiveness. Similar results were found by Doyle et al., where 85% of medical students agreed that it is difficult to question the decision or actions of those with more authority [16].

Medical errors are already the third leading cause of death in in the US hospitals, therefore education and training in patient safety needs to be a prerequisite in healthcare systems [23]. Though students observe many patient safety concerns during their internships, it is a missed opportunity that they do not address their concerns as the organization might learn and profit [16]. However, this might be difficult in a hierarchical organized system. A possible trigger in order not to lose the knowledge of students’ concern, students should be encouraged a) to speak up and b) as a time delayed alternative to use the critical incident reporting system. However, to achieve these goals, two things have to change in general.

On the one hand, the curriculum concept has to be adapted with regard to patient safety aspects. The growing importance of patient safety aspect requires medical education programs as already suggested by WHO and others [24,25]. The WHO Patient Safety Curriculum Guide for Medical Schools includes a manual for teachers on how to best integrate all patient safety relevant areas [26]. Priority areas in education of students should involve knowledge in cause and frequency of patient incidents, the willingness to take responsibility when it comes to patient harm, communication and teamwork skills for effective safe care and care delivery [27,28]. Nevertheless, a main challenge might be how to best combine content from medicine with human-factors and non-technical skills training to create an effective curriculum [29]. Teaching patient safety skills with an inter-professional approach heightened students’ awareness and importance of effective team-working [29]. One possible trigger could be that medical education profits through medical simulation as it was reported that intrinsic motivation of students significantly increase after simulation based training [30].

On the other hand, it is also necessary to establish a safety culture at the clinic. On the organizational level, academic clinical departments have a rich tradition of improving approaches such as prevention, diagnosis and treatment of disease, however, quality improvement and patient safety expertise has to be incorporated in academic clinical departments [24]. Furthermore, there is also a lack of senior or midcareer faculty members whose career have focused on quality improvement and patient safety who could then also act as positive role models for students and younger colleagues [24]. Therefore, for large-scale change to be successful in creating a culture of safety, a combination of a top-down approach, such as central coordination in educating HCPs and other healthcare providers, with bottom-up engagement where teams on the ground take control and ownership is needed [31].

This study has several limitations. A major limitation of this survey was that only 24% responded to the survey though students were reminded three times. One reason could have been that the survey was performed in May to June where students have to pass their exams or that not all students between the 5^th^ and 10^th^ term were recently exposed to a four weeks clinical term and skipped the survey. Moreover, the generalizability is significantly limited as the study was performed in a single institution in Austria. The study only focussed on medical students; however, it should be also of interest how nursing students during their internships perceive speaking up behaviours.

### Conclusions

Speaking up behaviours of students was assessed for the first time in a German speaking academic teaching hospital. Higher perceived risk of harm for the patient, past episodes of speaking up and a more favorable climate score were all significantly associated with a higher self-reported likelihood to speak up. All predefined barriers for not speaking up seemed to play an important role and was associated with a missing safety culture in attended clinical units during their internships. These results give hints that there is a need to adapt the curriculum concept with regard to patient safety aspects. Finally, there is also the need to establish a safety culture at the organizational and unit level. Speaking up can only work if HCPs and students perceive an open minded setting in their environment.
